# Experiences, perceptions and barriers to use of reusable menstrual products among university students globally: a systematic review

**DOI:** 10.1136/bmjopen-2025-103159

**Published:** 2025-08-06

**Authors:** Emma Johnson, Lydia Seed, Ashna Biju, Charlotte Tulinius

**Affiliations:** 1 University of Cambridge School of Clinical Medicine, Cambridge, UK; 2 Department of Public Health and Primary Care, Clinical School of Medicine, University of Cambridge, Cambridge, UK

**Keywords:** Health Education, Adolescent, PUBLIC HEALTH, Reproductive medicine

## Abstract

**Abstract:**

**Background:**

Reusable menstrual products have gained increasing attention for their environmental and economic benefits, yet their uptake remains limited. University students represent a key demographic for understanding uptake, as young adults forming lifelong menstrual hygiene habits.

**Objectives:**

To synthesise evidence on university students’ knowledge, usage patterns and perceptions of reusable menstrual products, and identify barriers and facilitators to their adoption, to inform future interventions and educational efforts.

**Search strategy:**

A systematic search of Medline, Embase, Scopus and Global Health was conducted, last updated on 31 October 2024.

**Selection criteria:**

Eligible studies contain data on perceptions of reusable menstrual products, specifically of students in university or higher education, or including segregated data on this population. Qualitative, quantitative and mixed-methods studies were included.

**Data collection and analysis:**

Two independent reviewers screened studies, extracted data and assessed methodological quality. All data were summarised descriptively.

**Results:**

10 studies (4721 participants) across multiple countries were included. Findings suggest that while reusable menstrual products are viewed by some as cost-effective and sustainable, barriers include concerns relating to practical usage and health, limited awareness, misconceptions and cultural taboos. In the university context, peer influence, financial constraints and sociocultural factors play a significant role in shaping product choices.

**Conclusions:**

Targeted education to increase awareness and address practical concerns and misconceptions, alongside peer support and provision of reusable menstrual products, could significantly enhance the adoption of reusable menstrual products in university settings. Further research into the health impacts of these products would support educational interventions.

**STRENGTHS AND LIMITATIONS OF THIS STUDY**

- A comprehensive search strategy was conducted across four major databases with no language, location or date restrictions, producing a globally diverse sample.
- This protocol adheres to the Preferred Reporting Items for Systematic Reviews and Meta-Analysis guidelines and study selection, data extraction, quality assessment and certainty assessment were conducted independently by multiple researchers, ensuring consistency and reliability.
- Most included studies focused primarily on menstrual cups, limiting insights into other reusable products.
- Limited qualitative data reduce the depth of insight into perceptions.

## Introduction

 Menstrual hygiene management is a public health, environmental and gender equality issue, and a critical component of personal health. It requires access to water, sanitation facilities and quality menstrual products.^1^ Such products range from disposable, single-use pads and tampons to reusable products including period underwear, washable pads and menstrual cups. Inadequate menstrual hygiene management can lead to missed educational or work opportunities, reinforcement of gender-based stigma and inequality,^1^ reproductive and urinary tract infections, mental health problems^2^ and plastic waste from disposable products.

In recent years, the use of reusable menstrual products has grown as a result of many factors, including their environmental and cost benefits compared with single-use products.^3^ The reusable sanitary pad market is projected to rapidly grow over the next 10 years, most significantly in the Asia-Pacific region.^4^ For example, India’s reusable menstrual product market is gaining momentum with increased interest from consumers, though reusable options still represent 1–2% of the total market.^5^ A growing body of research in low- and middle-income countries has highlighted the role of reusable products in improving menstrual health, reducing waste and alleviating period poverty.^3^ However, barriers to their widespread use globally remain, such as cultural norms, lack of awareness and misconceptions around their use, especially the menstrual cup.^6 7^

Lack of access to menstrual products is recognised globally to be a barrier to accessing education.^1^ To address this, many governments worldwide, including Australia, Kenya and France, provide access to free menstrual products in schools.^8^ The UK government launched the Period Product Scheme in 2020, providing free menstrual products for all pupils in schools and 16–19 education organisations.^9^ Scotland has advanced this further to apply to higher education institutions; however, this is not the case in the rest of the UK.^10^ Several universities are taking it upon themselves to independently provide free period products. For example, students at the University of Cambridge have successfully lobbied for free provision following a long period of campaigning.^11^ However, reusable period products are rarely included in free menstrual product provision programmes and current research assessing the effects of reusable menstrual products on school attendance is inconsistent.^3^ Further evaluation of the potential for reusable menstrual products to reduce this barrier to education is warranted.

Studies suggest that young adults are more environmentally conscious than the previous generation and want to engage in more sustainable consumer behaviours.^12^ It has also been suggested that university students may be more amenable to change before lifetime habits are established and are more open to peer influence.^13^ Therefore, the uptake of reusable menstrual products among university students could be high. Understanding perceptions of reusable period products is an important step to removing barriers and facilitating uptake among university students. This provides potential for research into perceptions of period products, especially reusable products, among university students.

To the best of our knowledge, no study has systematically examined the perceptions of all reusable menstrual products among university students globally. In the present study, we intend to address this gap in the literature by examining knowledge, usage patterns, perceptions and determinants of use of reusable menstrual products, particularly menstrual cups, among university and higher education students globally.

## Methods

**Figure 1:**
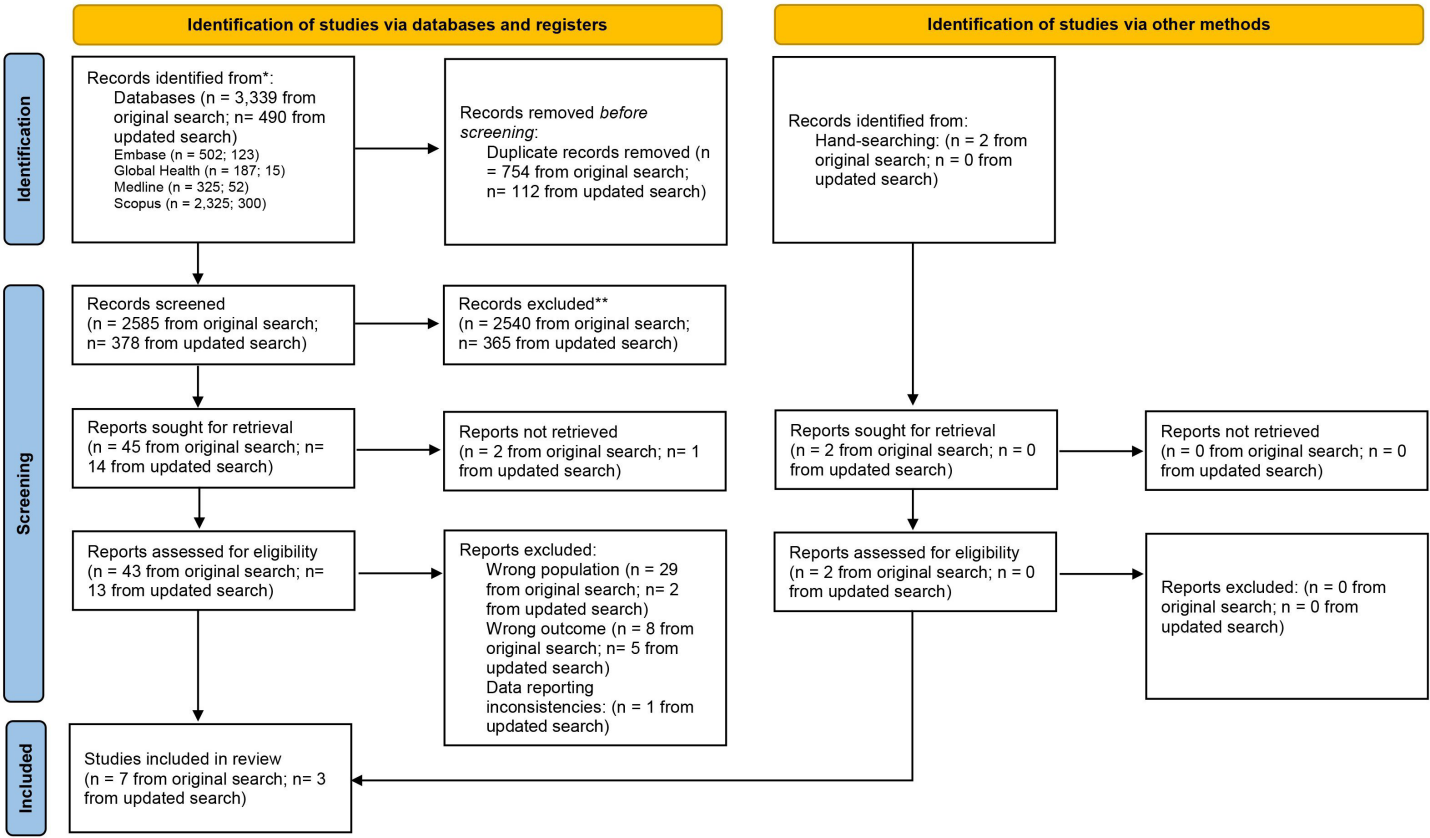
Preferred Reporting Items for Systematic Reviews and Meta-Analysis flowchart. *Consider, if feasible to do so, reporting the number of records identified from each database or register searched (rather than the total number across all databases/registers). **If automation tools were used, indicate how many records were excluded by a human and how many were excluded by automation tools. Source: Page *et al*. This work is licensed under CC BY 4.0. To view a copy of this licence, visit https://creativecommons.org/licenses/by/4.0/.

This systematic review is reported in accordance with the Preferred Reporting Items for Systematic Reviews and Meta-Analysis guidelines (figure 1).^14^ The review protocol was registered a priori in PROSPERO (CRD42023451664). A comprehensive literature search was conducted across Medline, Embase, Scopus and Global Health. Initial searches took place on 12 September 2023 (online supplemental material 1). There were no language or publication date restrictions. The search yielded 2585 articles after removal of duplicates. Title and abstract screening was performed independently by two reviewers (EJ and LS) using Rayyan. Four conflicts were resolved by discussion, leaving 45 potentially relevant articles. Full-text screening was conducted independently by the same two reviewers, with reasons for exclusion clearly documented. Three conflicts were resolved by discussion, leaving five articles for inclusion. Two additional papers were identified through manual searching. The search was repeated on 31 October 2024 to ensure up-to-date results. This yielded 378 articles after duplicate removal, leaving 13 after title and abstract screening. Full-text review required three conflicts to be resolved by discussion, leaving three papers to be included. This resulted in a total of 10 included studies.

### Study selection

Studies investigating university students’ perceptions of reusable menstrual products were included. ‘Perceptions’ were defined as ideas about and opinions of reusable menstrual products, as well as personal experiences with their use. Eligible studies focused solely on students in university or higher education, or include segregated data on this population. Studies involving only school-aged children or adults not in higher education, as well as those discussing only single-use menstrual products, were not of interest. There were no restrictions on study design; however, studies were required to provide data on perceptions of reusable menstrual products—studies reporting only numerical data on use of reusable products, without reporting perceptions, were excluded. Quantitative, qualitative and mixed-methods studies were included. Studies reported only in abstract form and those not peer-reviewed were excluded.

### Data extraction

A pilot data extraction form was tested by three reviewers (EJ, LS and AB) and subsequently refined. Data extracted included study characteristics, population characteristics, details about menstrual product use and perceptions. Data extraction was performed independently by a minimum of two reviewers per study, with discrepancies resolved through discussion.

#### Quality assessment

The quality of included studies was assessed using the Mixed Methods Appraisal Tool (MMAT)^15^ (online supplemental material 2). Each study was independently assessed by two reviewers (EJ, AB), with discrepancies resolved by discussion. The protocol initially specified the use of Critical Appraisal Skills Programme (CASP) checklists;^16^ however, five of the included were cross-sectional, for which there is not an appropriate CASP checklist—MMAT was therefore used.

#### Synthesis of findings

A narrative synthesis was performed. All data were summarised descriptively in table format, with studies ordered by publication year. Studies published in the same year were ordered alphabetically by lead author surname. Separate analyses were conducted for data on menstrual product use and perceptions of reusable menstrual products. A meta-analysis was not possible due to the heterogeneity of the data. Heterogeneity across studies was examined by considering difference in setting, population, study design and prior experience with reusable products. Given the absence of meta-analysis, the Synthesis without meta-analysis (SWiM) checklist^17^ was used to guide synthesis and reporting of findings. Confidence in review findings was assessed using the Grading of Recommendations Assessment, Development and Evaluation – Confidence in the Evidence from Reviews of Qualitative Research (GRADE-CERQual) approach^18^ by two reviewers (EJ, AB).

### Patient and public involvement

Patients and the public were not involved in the design, conduct, reporting or dissemination of this study.

## Results

### Study characteristics

Key study characteristics are summarised in table 1.

**Table 1:** Key characteristics of the included studies

Lead author, year/country	Study design	Sample size	Participant age	Study population	Study aims
Grose, 2014/USA^25^	Cross-sectional (questionnaire with one free text question)	151	Mean age: 19.33 Range: 18–23	Female undergraduates at large public university in California	Investigate attitudes and reactions towards the menstrual cup and consider any correlation with ethnicity or self-objectification
Huang, 2019/Taiwan^19^	Cross-sectional (online questionnaire)	1245	NR	Female students, aged >18, at various universities in Taiwan	Explore factors associated with menstrual cup use intention
Beksinska, 2021/South Africa^20^	Cohort (paper questionnaires and interviews at baseline, 1, 6 and 12 months. Menstrual cups given)	509 enrolled—follow-up: 91% at 1 month 87% at 6 months 32.4% at 12 months	Mean age: 21 Range: 18–24	Female students at 10 public further education institutions across KwaZulu-Natal	Evaluation of long-term user acceptability of menstrual cups
Ganz, 2022/South Africa^21^	Cross-sectional (online questionnaire)	178	Mean age: 21.5 Range: 18–30	Medical students in their clinical years at the University of Witwatersrand (15% male, 82% female)	Investigate understanding and perception of the menstrual cup among medical students
Lobascz, 2022/Brazil^22^	Cross sectional (online questionnaire)	164	Mean age: 22.26±3.21 Range: NR	Women who matriculated at Botucatu Medical School	Obtain prevalence of and identify determinants of menstrual cup use
Owen, 2022/Australia^27^	Cohort (menstrual cups given, followed by dual diary and interview)	11	Mean age: NR Range: 20–24	Female undergraduates (current or recent) at Monash University, Melbourne who had never used a menstrual cup	Investigate how contemporary reorganisation of the menstrual cup affected menstrual experience
Abraham, 2023/India^23^	Cross-sectional (questionnaire)	187	Mean age: 20.49±2.07 Range: NR	Female students at Sree Narayana Engineering and Arts College	Explore knowledge, acceptance and misconceptions of the menstrual cup
Bhanawat, 2023/India^24^	Cross-sectional (self-administered questionnaire)	256	Mean age: NR Range: 16–23	Female Medical Students at Banas Medical College and Research Institute	Assess how female medical students perceived and used menstrual cups
James, 2024/India^28^	Cohort (educational intervention)	83	Mean age: 19.5 Range: 18–24	Female students in a degree college in peri-urban Bangalore	Assess the impact of health educational intervention on perceptions of menstrual cup usage
Soumyaja, 2024/India^26^	Cross-sectional (questionnaire with multi-choice and open-ended)	1937	Mean age: 19.8 Range: 18–24	Menstruating undergraduate (1317) and postgraduate (620) college/university students in Kerala (across 23 colleges)	Understand awareness, usage, and intention to use menstrual cups, and reasons for reluctance or adoption of cups

NR, not reported.

Ten studies were included. Eight studies administered questionnaires, of which six were purely quantitative,^19^,^24^ while the other two employed mixed methods with both multiple-choice and open-ended questions.^25 26^ 3 of the 10 studies were cohort studies, of which 2 offered menstrual cups to participants, while the third assessed the impact of an educational intervention on perceptions of menstrual cups.^20 27 28^ The studies were conducted in diverse locations and a total of 4721 participants were included, with mean ages ranging from 19.33 to 22.26 (excluding three studies which did not report mean age).

### Knowledge and patterns of usage of reusable menstrual products

Six studies reported patterns of menstrual product use (table 2).^1920 22^,^24 26^

**Table 2:** Knowledge and patterns of usage of menstrual products

Lead author, year/country	Use of single-use sanitary pads	Use of single-use tampon	Use of menstrual cup	Use of other reusable products	Awareness of reusable menstrual products
Grose, 2014/USA^25^	NR	NR	NR	NR	33% had heard of the menstrual cup prior to the survey
Huang, 2019/Taiwan^19^	63.8% (n=794)	31.4% (n=391)	4.8% (n=60)	NR	NR
Beksinska, 2021/South Africa^20^	95.5% (n=486) disposable pads 65.0% (n=331) panty liner In 3 months before baseline survey	7.9% (n=40) in 3 months before baseline survey	0.4% (n=2/509) in 3 months before baseline survey 86% (n=398/493) had used the menstrual cup at 1 month follow-up 87.1% (n=386/443) had used the menstrual cup at 6 month follow-up 85.3% (n=133/156) had used the menstrual cup at 12 month follow-up	Washable pads: 0.6% (n=3) in 3 months before baseline survey	21.8% reported ever having heard of the menstrual cup before the training session
Ganz, 2022/South Africa^21^	NR	NR	NR	NR	58.93% had a basic understanding of the menstrual cup as a menstrual hygiene product Females were 7.467 x more likely to have heard of the menstrual cup than males
Lobascz, 2022/Brazil^22^	82.93% (n=136) in last 12 months	36.59% (n=60) in last 12 months	17.07% (n=28) in last 12 months 22.56% (n=37) ever used	NR	NR
Owen, 2022/Australia^27^	NR	NR	To be included in the study participants must have never used a menstrual cup prior to the study	NR	NR
Abraham, 2023/India^23^	90.4% (n=169) at time of study	NR	1.07% (n=2) regular use for >1 year 4.81% (n=9) occasional use	NR	96.3% had heard of the menstrual cup
Bhanawat, 2023/India^24^	NR	NR	3.91% (n=10/256)	NR	85.94% (n=220/256) have heard of the menstrual cup
James, 2024/India^28^	NR	NR	NR	NR	72.3% (n=60/83) had heard of the menstrual cup at baseline. 100% after the intervention.
Soumyaja, 2024/India^26^	61.33% (n=1188/1937)	0.2% (n=4/1937)	7.65% (n=148/1927)	Cotton cloth or cloth pads: 30.77% (n=596/1937) Menstrual underwear: 0.05% (n=1/1937)	100% aware of cotton/cloth pads 85% aware of menstrual cup 2% aware of menstrual underwear

NR, not reported.

Single-use sanitary pads were the most commonly used product, ranging from 61.33% to 90.4%. The reusable menstrual product most commonly studied was the menstrual cup, although its use at baseline was low in all studies. Awareness of the menstrual cup varied considerably. Only two studies examined use of a reusable menstrual product other than the menstrual cup, namely washable pads, which were used by less than 1% of participants in these studies.^20 26^

### Quality assessment and confidence in review findings

Overall methodological quality was moderate to high in included studies. Common limitations included risk of non-response bias and lack of qualitative and quantitative integration in mixed methods studies (online supplemental material 2). One study was excluded from analysis due to low methodological quality with significant inconsistencies in data reporting, thus only studies with a low risk of bias were selected for the main synthesis.

Confidence in review findings varied: one finding was assessed as high confidence, four as moderate and one as low (online supplemental material 5). Our main concerns were methodological limitations, adequacy and relevance of the data. Concerns with adequacy included limited papers contributing to a finding of lack of richness of the data. The data were sometimes assessed as partially relevant due to studies focussing on subsets of university students, such as only medical students.

### Perceptions and determinants of use of reusable menstrual products

Perceptions and determinants of use of reusable menstrual products varied (online supplemental material 3), and studies reported perceptions from participants who had and had not used reusable menstrual products.

One study reported that 78% of participants were unwilling to use the menstrual cup,^23^ another found an ‘average’ intention of use,^26^ while another found, across all follow-up points, that 93–95% would recommend the menstrual cup to family members or friends.^20^ Satisfaction with current menstrual product was reported as an inhibiting factor for trying the menstrual cup.^23 24^

Education on how to use the menstrual cup was generally low, with 67.4%–74.4% having received no health education about the menstrual cup.^22 23^ Lack of knowledge was generally correlated with hesitancy and negative perceptions. However, interventions involving education or provision of a menstrual cup increased the number of participants with positive perceptions of menstrual cups in comparison to single-use products.^20 27 28^

Five studies discussed sustainability in relation to the menstrual cup, and its influence on cup use varied markedly. For many participants in some studies, sustainability was an important motivator for using the cup;^22 27^ however, in other studies, this was only important for a minority.^24 26^ In one study, 77.1% of participants were unaware of the menstrual cup as a sustainable product.^28^

Seven studies described financial implications of reusable menstrual products. Four found that participants viewed the menstrual cup as cost-effective, which motivated use for many.^19^,^2127^ In the other studies, less than one-quarter of participants perceived it as an important benefit or a determinant of use.^24^,^26^

Participants reported varied perceptions of the influence of the menstrual cup on physical health. Positive perceptions included promotion of vaginal health, and rash-free and itch-free periods.^22 24^ Negative perceptions included concerns about urinary problems, infections and allergies.^23 24 28^ In all papers except one,^28^ the proportion of participants concerned by physical health problems was <25%.

Studies also suggest that sociocultural factors heavily influenced perceptions of reusable menstrual products, including the effects of stigma, sexuality and feminism. Some participants saw the menstrual cup as ‘cool’, cited feminism as a reason to try the cup and felt its use invited more conversation and thought about menstruation.^27^ On the other hand, concerns about the menstrual cup causing loss of virginity and being unsuitable for married women were reported,^20 28^ and those with higher levels of self-objectification had more negative perceptions of the menstrual cup.^25^ Sexuality also influenced intended menstrual cup use with bisexual participants having higher intention rates and lesbians the lowest.^19^

The influence of menstrual flow on menstrual cup use was inconsistent; heavy flow was reported as a barrier to use,^22 23^ as well as a motivator among those with higher use intention.^19^ Light menstrual flow was also reported as a reason for not having tried the menstrual cup.^20^

## Discussion

This review provides insights into university students’ perceptions of reusable menstrual products, particularly menstrual cups. While environmental and financial benefits of reusable products are generally recognised, practical challenges, lack of knowledge and experience, and sociocultural factors remain significant barriers for some. The heterogeneity of reported perceptions highlights the need for tailored education and intervention efforts, including availability of a variety of products to suit individual preferences. These findings are supported by moderate to high methodological quality in all included studies and the certainty assessment found moderate to high confidence in all but one review finding.

No included studies reported negative views about financial implications, aligning with findings in school-children who perceive reusable products as financially beneficial.^29^ Concerns about upfront costs of reusable products have been reported more widely,^30^ but reusable product use can lead to significant savings over time compared with single-use products.^3^ However, the proportion of participants reporting financial benefit as a driver of use varied from 6% to 85.31%. This significant discrepancy may be explained by variation in experience of participants with the menstrual cup: studies with lower proportions of participants who had used a menstrual cup reported financial benefit to be less of a driver of use. There is also no standardised measure of ‘financial benefit’ which may explain these inconsistencies. Given that university students can experience period product insecurity,^11^ addressing affordability is essential among those for whom it is a concern.

Five studies in this review explored environmental concerns, yet only two found sustainability to be a significant motivator of reusable product use.^22 27^ The other studies found low awareness of the menstrual cup as a sustainable option and a low proportion of participants reporting this as a benefit.^24 26 28^ Wider research among young adults finds environmental concern to be a more significant motivator than in our included studies.^30 31^ This suggests increased awareness of benefits may improve uptake, and therefore that environmental messaging could be an effective component of interventions targeting university students.

Low levels of education on reusable menstrual products were reported, although few studies provided data on this, and a lack of knowledge about menstrual cups was linked to lower likelihood of use. This emphasises the importance of comprehensive educational initiatives which have been shown to improve attitude towards the menstrual cup.^28^ Educational initiatives may be most effective if they focus on peer support: one included study found menstrual cup use encouraged open conversations about menstruation, and many participants cited feminist peer groups as a reason for adopting the cup.^27^ Similarly, menstrual cup uptake among schoolgirls increased with peer-group education.^32^

Practical concerns were frequently cited barriers. Notably, concerns about leakage were frequently reported; however, in the wider literature, menstrual cup leakage is reported as similar or lower than that for disposable pads or tampons, across all ages.^6 33 34^ Concerns about leakage, insertion, removal or discomfort were less significant in those with experience of using the cup and those with more knowledge of the menstrual cup, either through direct experience or receiving education, tended to evaluate it more positively. Similar findings have been replicated in studies where schoolchildren were given menstrual cups—perceptions of the practicalities of using a menstrual cup improved with direct experience.^32 34^ This suggests that provision of menstrual cups to provide practical experience, alongside education in their use, could be a highly effective intervention.

Additionally, some found the menstrual cup convenient, minimising the impact of menstruation on daily activities,^27^ whereas others had concerns about changing the menstrual cup while away from home.^19 20^ This concern is echoed among other adults.^31^ Ensuring public toilets have adequate facilities to change reusable menstrual products while not at home is another intervention universities could consider to remove barriers to their use.

Comfort with vaginal insertion is essential for menstrual cup use—participants were more likely to choose the menstrual cup if they were not concerned with manipulation of the internal genitalia or virginity loss.^20 22^ Additionally, while some students found the direct encounter with menstrual blood empowering,^27^ others were deterred by it.^25^ This highlights the importance of awareness of alternative reusable menstrual products, such as period underwear that absorb menstrual blood and do not require vaginal insertion.

Concerns about the safety of the menstrual cup, such as the potential for it to cause allergies or infections, were reported.^23 24 26 28^ Other participants cited positive health impacts of the menstrual cup as reasons for adoption, such as itch-free or rash-free periods or vaginal health.^22 28^ Current available evidence suggests that menstrual cup use is safe and is not associated with increased risk of infections or mechanical harm to the vagina or cervix.^6 35^ However, there have been a small number of cases of toxic shock syndrome, vaginal wounds, allergies or rashes and urinary tract complaints after use of the menstrual cup.^6^ Further research is needed to establish the rates of these complications, so that robust comparisons can be made between reusable and single-use products. This will ensure users are fully informed to make choices regarding menstrual product selection and can be educated on the safest way to use them. This could fall within the scope of the Women’s Health Strategy for England,^36^ with menstrual health and gynaecological conditions identified as a priority area.

### Implications for practice

To facilitate the use of reusable menstrual products among university students, several implications for practice emerge from the study herein. Ensuring widespread access to reusable products is essential—providing such products has been shown to substantially increase usage and satisfaction.

Moreover, fear, discomfort and health concerns remain significant barriers to the use of reusable menstrual products. Addressing these negative perceptions is vital to encouraging wider adoption. This will require further research into the health impacts of these products, as well as exploring potential solutions to existing challenges users face. In addition, enhanced education is essential to promote confidence in reusable menstrual products. Increased awareness of other benefits through education, such as sustainability, may also promote uptake.

Additionally, menstrual cups may not be suitable for all users, particularly those with concerns about vaginal insertion of a product. Expanding research into other reusable products could help inform interventions and promote their accessibility more widely.

Finally, while reusable products are generally considered cost-effective long-term, the upfront cost can still be a barrier for some users, especially within university settings. Targeted financial assistance or subsidies may alleviate this issue.

### Strengths and limitations of this review

This is the first systematic review to assess perceptions of reusable menstrual products among university students, addressing a significant gap in the literature. One notable strength is the inclusion of studies from a diverse sample of countries, providing a global perspective on the topic. Although cultural differences may limit the generalisability of the conclusions, the findings present a broad array of perceptions.

However, there are several limitations. Most studies did not report disaggregated data based on whether participants had used the menstrual cup, limiting the interpretation of findings. Since most participants had not used the menstrual cup, the results predominantly reflect barriers to adoption and the perspectives of those with experience using the cup may be underrepresented.

The narrow focus on menstrual cups, while providing some generalisable views as well as product-specific insights, means that other reusable menstrual products have not been as rigorously investigated. Future research should explore perceptions of a broader range of products to provide a more comprehensive understanding.

A further limitation is the lack of qualitative data and free-text responses in questionnaires, which may restrict the depth of insight into factors influencing participants’ perceptions. Future studies could employ qualitative methods to explore in more detail the reasons behind people’s opinions. This is crucial for designing effective educational and intervention programmes.

## Conclusions

This systematic review presents a wide range of perceptions of reusable menstrual products, particularly menstrual cups, among university students across the globe.

While the environmental and financial benefits of reusable products are acknowledged, practical and physical health concerns such as discomfort, insertion and cleaning remain significant barriers to use. Cultural and socioeconomic factors, including concerns about virginity and menstrual taboos, also play a critical role in influencing attitudes towards these products across differing populations. Negative perceptions could be explained by the extent of knowledge, misinformation and availability of experience.

Strategies to increase use of reusable menstrual products could include comprehensive education programmes, coupled with provision of a product. Our findings also highlight the importance of peer-support programmes in promoting use. These initiatives must be context-specific to ensure inclusivity: considering cultural differences, affordability and access to menstrual products and infrastructure such as washing facilities to ensure efficacy. Further research to assess the health impacts of reusable products will assist in combating misinformation and educational initiatives. Expansion of research to include other reusable menstrual products is warranted to evaluate products that may better match the preferences of a diverse range of individuals. Usage of standardised outcome measures in this research would address current inconsistencies in how perceptions and usage are reported, and adding qualitative elements will increase depth of understanding.

## Supplementary material

10.1136/bmjopen-2025-103159 online supplemental file 1

10.1136/bmjopen-2025-103159 online supplemental file 2

10.1136/bmjopen-2025-103159 online supplemental file 3

10.1136/bmjopen-2025-103159 online supplemental file 4

## Data Availability

All data relevant to the study are included in the article or uploaded as supplementary information.
