# Supplementary material for: Experiences, perceptions and barriers to use of reusable menstrual products among university students globally: a systematic review
Source: BMJ Open. 2025 Aug 6;15(8):e103159. doi: 10.1136/bmjopen-2025-103159 (PMC12336580; doi:10.1136/bmjopen-2025-103159)
Supplement: online supplemental file 1 [file bmjopen-15-8-s001.pdf]

Database: Ovid MEDLINE(R) and Epub Ahead of Print, In-Process, In-Data-Review & Other Non-Indexed Citations, Daily and Versions <1946 to September 11, 2023>

Search Strategy:

1 "period underwear".ti,ab. (4)
2 "reusable pad*".ti,ab. (16)
3 "reusable".ti,ab. (8984)
4 "menstrual".ti,ab. (46714)
5 "period".ti,ab. (1555910)
6 "college".ti,ab. (139362)
7 "higher education".ti,ab. (19754)
8 "student".ti,ab. (108760)
9 "washable pad*".ti,ab. (2)
10 "reusable sanitary towel*".ti,ab. (1)
11 "sustainable".ti,ab. (101074)
12 "reusable tampon*".ti,ab. (0)
13 "universit*".ti,ab. (470417)
14 "menstrual cup".ti,ab. (79)
15 1 or 2 or 9 or 10 or 12 or 14 (97)
16 Menstrual Hygiene Products/ (638)
17 Universities/ (52804)
18 4 or 5 or 16 (1595216)
19 3 or 11 (109690)
20 18 and 19 (6333)
21 15 or 20 (6408)
22 6 or 7 or 8 or 13 or 17 (692533)
23 21 and 22 (325)

Database: Embase <1974 to 2023 September 11>

Search Strategy:

1 "period underwear".ti,ab. (2)

2 "reusable pad*".ti,ab. (5)

3 "reusable".ti,ab. (11182)

4 "menstrual".ti,ab. (60899)

5 "period".ti,ab. (2179965)

6 "college".ti,ab. (265223)

7 "higher education".ti,ab. (23528)

8 "student".ti,ab. (144550)

9 "washable pad*".ti,ab. (2)

10 "reusable sanitary towel*".ti,ab. (0)

11 "sustainable".ti,ab. (103176)

12 "reusable tampon*".ti,ab. (0)

13 "universit*".ti,ab. (807810)

14 "menstrual cup".ti,ab. (112)

15 1 or 2 or 9 or 10 or 12 or 14 (117)

16 feminine hygiene product/ (308)

17 university/ (120956)

18 4 or 5 or 16 (2230316)

19 3 or 11 (113962)

20 18 and 19 (7535)

21 15 or 20 (7635)

22 6 or 7 or 8 or 13 or 17 (1179455)

23 21 and 22 (495)

#50	final	(((TITLE-ABS-KEY ("period underwear")) OR (TITLE-ABS-KEY ("reusable pad")) OR (TITLE-ABS-KEY ("reusable sanitary towel")) OR (TITLE-ABS-KEY ("washable pads")) OR (TITLE-ABS-KEY ("menstrual cup"))) OR (((TITLE-ABS-KEY (menstrual)) OR (TITLE-ABS-KEY (period))) AND ((TITLE-ABS-KEY (sustainable)) OR (TITLE-ABS-KEY (reusable))))) AND ((TITLE-ABS-KEY (college)) OR (TITLE-ABS-KEY (student)) OR (TITLE-ABS-KEY (universit*)) OR (TITLE-ABS-KEY ("higher education"))) View Less Edit query	2,293	02 Aug 2023
#49	college student universit high	(TITLE-ABS-KEY (college)) OR (TITLE-ABS-KEY (student)) OR (TITLE-ABS-KEY (universit*)) OR (TITLE-ABS-KEY ("higher education")) View Less Edit query	3,383,468	02 Aug 2023
#48	period underwear reusable pad	((TITLE-ABS-KEY ("period underwear")) OR (TITLE-ABS-KEY ("reusable pad")) OR (TITLE-ABS-KEY ("reusable sanitary towel")) OR (TITLE-ABS-KEY ("washable pads")) OR (TITLE-ABS-KEY ("menstrual cup"))) OR (((TITLE-ABS-KEY (menstrual)) OR (TITLE-ABS-KEY (period))) AND ((TITLE-ABS-KEY (sustainable)) OR (TITLE-ABS-KEY (reusable)))) View Less Edit query	48,545	02 Aug 2023
#47	menstrual period sustainable r	((TITLE-ABS-KEY (menstrual)) OR (TITLE-ABS-KEY (period))) AND ((TITLE-ABS-KEY (sustainable)) OR (TITLE-ABS-KEY (reusable))) View Less Edit query	48,420	02 Aug 2023

#46	sustainable reusable	(TITLE-ABS-KEY (sustainable)) OR (TITLE-ABS-KEY (reusable))	822,532	02 Aug 2023
#45	menstrual period	(TITLE-ABS-KEY (menstrual)) OR (TITLE-ABS-KEY (period))	4,357,009	02 Aug 2023
#44	products	(TITLE-ABS-KEY ("period underwear")) OR (TITLE-ABS-KEY ("reusable pad")) OR (TITLE-ABS-KEY ("reusable sanitary towel")) OR (TITLE-ABS-KEY ("washable pads")) OR (TITLE-ABS-KEY ("menstrual cup")) View Less Edit query	173	02 Aug 2023
#43	menstrual cup	TITLE-ABS-KEY ("menstrual cup")	149	02 Aug 2023
#42	higher education	TITLE-ABS-KEY ("higher education")	194,566	02 Aug 2023
#41	washable pads	TITLE-ABS-KEY ("washable pads")	2	02 Aug 2023
#40	reusable sanitary towel	TITLE-ABS-KEY ("reusable sanitary towel")	1	02 Aug 2023
#39	reusable pad	TITLE-ABS-KEY ("reusable pad")	27	02 Aug 2023
#38	period underwear	TITLE-ABS-KEY ("period underwear")	3	02 Aug 2023
#29	universit	TITLE-ABS-KEY (universit*)	1,887,879	01 Aug 2023
#27	sustainable	TITLE-ABS-KEY (sustainable)	775,477	01 Aug 2023
#24	student	TITLE-ABS-KEY (student)	1,549,190	01 Aug 2023
#22	college	TITLE-ABS-KEY (college)	511,586	01 Aug 2023
#21	period	TITLE-ABS-KEY (period)	4,291,199	01 Aug 2023
#20	menstrual	TITLE-ABS-KEY (menstrual)	80,172	01 Aug 2023
#19	reusable	TITLE-ABS-KEY (reusable)	48,947	01 Aug 2023

			Database - Global Health	
S15	DE "menstrual cycle" OR DE "menstruation"	Expanders - Apply equivalent subjects<br>Search modes - Boolean/Phrase	Interface - EBSCOhost Research Databases<br>Search Screen - Advanced Search<br>Database - Global Health	5,152
S14	TI (menstrual cup) OR AB (menstrual cup)	Expanders - Apply equivalent subjects<br>Search modes - Boolean/Phrase	Interface - EBSCOhost Research Databases<br>Search Screen - Advanced Search<br>Database - Global Health	34
S13	TI (universit*) OR AB (universit*)	Expanders - Apply equivalent subjects<br>Search modes - Boolean/Phrase	Interface - EBSCOhost Research Databases<br>Search Screen - Advanced Search<br>Database - Global Health	96,431
S12	TI (reusable tampon*) OR AB (reusable tampon*)	Expanders - Apply equivalent subjects<br>Search modes - Boolean/Phrase	Interface - EBSCOhost Research Databases<br>Search Screen - Advanced Search<br>Database - Global Health	1
S11	TI (sustainable) OR AB (sustainable)	Expanders - Apply equivalent subjects<br>Search modes - Boolean/Phrase	Interface - EBSCOhost Research Databases<br>Search Screen - Advanced Search<br>Database - Global Health	37,115
S10	TI (reusable sanitary towel*) OR AB (reusable sanitary towel*)	Expanders - Apply equivalent subjects<br>Search modes - Boolean/Phrase	Interface - EBSCOhost Research Databases<br>Search Screen - Advanced Search<br>Database - Global Health	0
S9	TI (washable pad*) OR AB (washable pad*)	Expanders - Apply equivalent subjects<br>Search modes - Boolean/Phrase	Interface - EBSCOhost Research Databases<br>Search Screen - Advanced Search<br>Database - Global Health	1
S8	TI (student) OR AB (student)	Expanders - Apply equivalent subjects<br>Search modes - Boolean/Phrase	Interface - EBSCOhost Research Databases<br>Search Screen - Advanced Search<br>Database - Global Health	77,533
S7	TI (higher education) OR AB (higher education)	Expanders - Apply equivalent subjects<br>Search modes - Boolean/Phrase	Interface - EBSCOhost Research Databases<br>Search Screen - Advanced Search<br>Database - Global Health	10,948
S6	TI (college) OR AB (college)	Expanders - Apply equivalent subjects<br>Search modes - Boolean/Phrase	Interface - EBSCOhost Research Databases<br>Search Screen - Advanced Search<br>Database - Global Health	28,181
S5	TI (period) OR AB (period)	Expanders - Apply equivalent subjects<br>Search modes - Boolean/Phrase	Interface - EBSCOhost Research Databases<br>Search Screen - Advanced Search<br>Database - Global Health	348,879
S4	TI (menstrual) OR AB (menstrual)	Expanders - Apply equivalent subjects<br>Search modes - Boolean/Phrase	Interface - EBSCOhost Research Databases<br>Search Screen - Advanced Search<br>Database - Global Health	6,707
S3	TI (reusable) OR AB (reusable)	Expanders - Apply equivalent subjects<br>Search modes - Boolean/Phrase	Interface - EBSCOhost Research Databases<br>Search Screen - Advanced Search<br>Database - Global Health	1,051
S2	TI (reusable pad*) OR AB (reusable pad*)	Expanders - Apply equivalent subjects<br>Search modes - Boolean/Phrase	Interface - EBSCOhost Research Databases<br>Search Screen - Advanced Search<br>Database - Global Health	25
S1	TI (period underwear) OR AB (period underwear)	Expanders - Apply equivalent subjects<br>Search modes - Boolean/Phrase	Interface - EBSCOhost Research Databases<br>Search Screen - Advanced Search<br>Database - Global Health	1

#	Query	Limiters/Expanders	Last Run Via	Results
S26	S24 AND S25	Expanders - Apply equivalent subjects<br>Search modes - Boolean/Phrase	Interface - EBSCOhost Research Databases<br>Search Screen - Advanced Search<br>Database - Global Health	187
S25	S6 OR S7 OR S8 OR S13 OR S16 OR S17 OR S18	Expanders - Apply equivalent subjects<br>Search modes - Boolean/Phrase	Interface - EBSCOhost Research Databases<br>Search Screen - Advanced Search<br>Database - Global Health	179,096
S24	S20 OR S23	Expanders - Apply equivalent subjects<br>Search modes - Boolean/Phrase	Interface - EBSCOhost Research Databases<br>Search Screen - Advanced Search<br>Database - Global Health	3,350
S23	S21 AND S22	Expanders - Apply equivalent subjects<br>Search modes - Boolean/Phrase	Interface - EBSCOhost Research Databases<br>Search Screen - Advanced Search<br>Database - Global Health	3,323
S22	S3 OR S11 OR S19	Expanders - Apply equivalent subjects<br>Search modes - Boolean/Phrase	Interface - EBSCOhost Research Databases<br>Search Screen - Advanced Search<br>Database - Global Health	44,838
S21	S4 OR S5 OR S15	Expanders - Apply equivalent subjects<br>Search modes - Boolean/Phrase	Interface - EBSCOhost Research Databases<br>Search Screen - Advanced Search<br>Database - Global Health	355,664
S20	S1 OR S2 OR S9 OR S10 OR S12 OR S14	Expanders - Apply equivalent subjects<br>Search modes - Boolean/Phrase	Interface - EBSCOhost Research Databases<br>Search Screen - Advanced Search<br>Database - Global Health	57
S19	DE "sustainability"	Expanders - Apply equivalent subjects<br>Search modes - Boolean/Phrase	Interface - EBSCOhost Research Databases<br>Search Screen - Advanced Search<br>Database - Global Health	16,139
S18	DE "students"	Expanders - Apply equivalent subjects<br>Search modes - Boolean/Phrase	Interface - EBSCOhost Research Databases<br>Search Screen - Advanced Search<br>Database - Global Health	25,366
S17	DE "higher education"	Expanders - Apply equivalent subjects<br>Search modes - Boolean/Phrase	Interface - EBSCOhost Research Databases<br>Search Screen - Advanced Search<br>Database - Global Health	1,365
S16	DE "college students"	Expanders - Apply equivalent subjects<br>Search modes - Boolean/Phrase	Interface - EBSCOhost Research Databases<br>Search Screen - Advanced Search<br>Database - Global Health	11,068
S15	DE "menstrual cycle" OR DE "menstruation"	Expanders - Apply equivalent subjects<br>Search modes - Boolean/Phrase	Interface - EBSCOhost Research Databases<br>Search Screen - Advanced Search<br>Database - Global Health	5,152
S14	TI (menstrual cup) OR AB (menstrual cup)	Expanders - Apply equivalent subjects<br>Search modes - Boolean/Phrase	Interface - EBSCOhost Research Databases<br>Search Screen - Advanced Search<br>Database - Global Health	34
S13	TI (universit*) OR AB (universit*)	Expanders - Apply equivalent subjects<br>Search modes - Boolean/Phrase	Interface - EBSCOhost Research Databases<br>Search Screen - Advanced Search<br>Database - Global Health	96,431
S12	TI (reusable tampon*) OR AB (reusable tampon*)	Expanders - Apply equivalent subjects<br>Search modes - Boolean/Phrase	Interface - EBSCOhost Research Databases<br>Search Screen - Advanced Search<br>Database - Global Health	1
S11	TI (sustainable) OR AB (sustainable)	Expanders - Apply equivalent subjects<br>Search modes - Boolean/Phrase	Interface - EBSCOhost Research Databases<br>Search Screen - Advanced Search	37,115
