# Supplementary material for: Experiences, perceptions and barriers to use of reusable menstrual products among university students globally: a systematic review
Source: BMJ Open. 2025 Aug 6;15(8):e103159. doi: 10.1136/bmjopen-2025-103159 (PMC12336580; doi:10.1136/bmjopen-2025-103159)
Supplement: online supplemental file 2 [file bmjopen-15-8-s002.docx]

**Supplementary Material 2**: Methodological Quality of the Data using MMAT^15^

|  | **Are there clear research questions?** | **Do collected data allow to address research questions?** | **MMAT Category 4: Quantitative Descriptive** | Is the sampling strategy relevant to address the research question? | Is the sample representative of the target population? | Are the measurements appropriate? | Is the risk of non-response bias low? | Is the statistical analysis appropriate to answer the research question? |
| --- | --- | --- | --- | --- | --- | --- | --- | --- |
| **Huang, 2019 / Taiwan^20^** | Yes | Yes |  | Yes | Yes | Yes | Can’t tell | Yes |
| **Ganz, 2022 / South Africa^22^** | Yes | Yes |  | Yes | Can’t tell | Yes | Can’t tell | Yes |
| **Lobascz, 2022 / Brazil^23^** | Yes | Yes |  | Yes | Yes | Yes | No (59.2% response rate to online survey invitation) | Yes |
| **Abraham, 2023 / India^25^** | Yes | Yes |  | Yes | Yes | Yes | Yes | Yes |
| **Bhanawat, 2023 / India^26^** | Yes | Yes |  | Yes | No (only study medical students, target is adolescents) | No (questionnaire ‘validation’ based on check by PI) | Yes (77% response rate to online survey) | Yes |
|  |  |  | **MMAT Category 3: Quantitative non-randomised** | Are the participants representative of the target population? | Are measurements appropriate regarding both the outcome and intervention (or exposure)? | Are there complete outcome date? | Are the confounders accounted for in the design and analysis? | During the study period, is the intervention administered (or exposure occurred) as intended? |
| **Beksinska, 2021 / South Africa^21^** | Yes | Yes |  | Yes | Yes (baseline questionnaire, follow up surveys and interviews at 1, 6 and 12 months) | No (Follow up at 12 months only 32.4%) | No (convenience sampling, not stratified to sociodemographic data) | Yes |
| **James, 2024 / India^27^** | Yes | Yes |  | Yes | Yes | Yes | No (convenience sampling, not stratified to sociodemographic data) | Yes |
|  |  |  | **MMAT Category 5: Mixed Methods** | Is there an adequate rationale for using a mixed methods design to address the research question? | Are the different components of the study effectively integrated to answer the research question? | Are the outputs of the integration of qualitative and quantitative components adequately interpreted? | Are divergences and inconsistencies between quantitative and qualitative results adequately addressed? | Do the different components of the study adhere to the quality criteria of each tradition of the methods involved? |
| **Grose, 2014 / USA^19^** | Yes | Yes |  | No (no rationale given) | No (no explicit integration  - quantitative questionnaire with single open-ended question) | No (no explicit integration) | Can’t tell (only 1 qualitative Q, no divergences to account for) | No (quantitative aspect adequate, qualitative question does not adhere to criteria) |
| **Soumyaja, 2024 / India^28^** | Yes | Yes |  | Yes (open-ended questions to understand reasons behind answers to quantitative questions) | Yes | Yes | Yes | Yes |
|  |  |  | **MMAT Category 1: Qualitative** | Is the qualitative approach appropriate to answer the research question? | Are the qualitative data collection methods adequate to address the research question? | Are the findings adequately derived from the data? | Is the interpretation of results sufficiently substantiated by data? | Is there coherence between qualitative data sources, collection, analysis and interpretation? |
| **Owen, 2022 / Australia^24^** | Yes | Yes |  | Yes | Yes (dual diary and interview technique) | Yes | Yes | Yes |

One paper was excluded due to significant inconsistencies in data reporting:

1. Jamkhandi SS, Tile R. Awareness, Acceptability, and Feasibility of the Menstrual Cup: A Descriptive Study. J South Asian Feder Obst Gynae 2024;16(4):346–349.
