# Supplementary material for: Experiences, perceptions and barriers to use of reusable menstrual products among university students globally: a systematic review
Source: BMJ Open. 2025 Aug 6;15(8):e103159. doi: 10.1136/bmjopen-2025-103159 (PMC12336580; doi:10.1136/bmjopen-2025-103159)
Supplement: online supplemental file 3 [file bmjopen-15-8-s003.docx]

**Supplementary Material 3.** Perceptions and determinants of use of reusable menstrual products

| **Lead Author, Year / Country** | **General Comments** | **Practicalities of using the menstrual cup** | **Influence of heavy menstrual bleeding** | **Lack of knowledge** | **Environmental concern** |
| --- | --- | --- | --- | --- | --- |
| **Grose, 2014 / USA^19^** | - 13.9% probably or definitely would use the cup if provided to them  - 14% (n=21) rejected the entire concept of the menstrual cup, including a participant stating “everything [should change]”  - 15.9% would recommend the menstrual cup to a friend or acquaintance | - n=72 (48%) concerned re presumed discomfort, reporting should be smaller, thinner or different shape  - n=15 (10%) concerned menstrual cup seemed difficult  to use (functional difficulty) and might leak |  | Participants who had heard of the menstrual cup before the study evaluated it more positively |  |
| **Huang, 2019 / Taiwan^20^** |  | Participants were less likely to try to use a menstrual cup if:  - they believed that it requires regular disinfection and cleaning  - they believe it is inconvenient to change the cup whilst going out  - they would like to engage in water activities during menstruation | - 56.4% of college girls with heavy menstrual flow had high menstrual cup use intention  - 47.1% of those with light menstrual flow had high menstrual cup use intention  - 40.6% of those with normal menstrual flow had high menstrual cup use intention | Participants were less likely to try to use a menstrual cup if they:  - had a lack of knowledge about how to use the cup  - had a lack of knowledge about the correct size of cup |  |
| **Beksinska, 2021 / South Africa^21^** | - across all follow up points 93-95% would recommend the menstrual cup to family members or friends | Reasons students accepted the menstrual cup at baseline:  - 16.9%: better protection  - 3.3%: ease of use at college  At 1 month follow up, of those who used the menstrual cup:  - 49.5% reported first insertion as very or quite easy  - 82.8% of those who did not find insertion very easy felt insertion improved with practice  - 71.1% reported removal was very or quite easy  - 78.3% of those who did not find removal very easy, felt it improved with practice  Of those who reported problems with insertion:  - pain / discomfort: 45.6%  - difficulty keeping cup folded / compressed: 26%  - difficulty with internal positioning of cup: 18.8%  - afraid / scared: 11.6%  Of those who reported problems with removal:  - pain / discomfort: 35%  - difficulty breaking suction: 30.6%  - contents spilled: 21.1%  - difficulty finding appropriate facility for removal / cleaning: 3.2%  - afraid / scared: 18.1%  At 1 month follow up, of the 14% who did not try to use the menstrual cup:  - 44.6% said they were afraid to try it  - 15.4% said insertion was uncomfortable or painful  Roughly 50% at each follow up reported choosing to go to their student residence / home to clean the cup, or only using it at weekends | - light menstrual flow was reported as a reason for having not tried the menstrual cup (participants reported that they were waiting for a heavier menstrual cycle to try it) |  |  |
| **Ganz, 2022 / South Africa^22^** | - 63.98% of medical students would recommend the menstrual cup to their future patients | ‘The menstrual cup is more convenient to use than other MHPs’  - 34.12% agreed/strongly agreed  - 33.18% disagreed/strongly disagreed  ‘The menstrual cup is more uncomfortable than alternative menstrual hygiene products’  - 37.56% disagree  - 20.66% agree  - 41.78% neutral  ‘It is difficult to remove and clean the menstrual cup’  - 46% disagree (significant proportion)  - 24% agree  - 30% neutral  - “the consensus as to whether the menstrual cup is difficult to insert remained equivocal” |  |  |  |
| **Lobascz, 2022 / Brazil^23^** |  | - 17.07% (n=28/164) reported any problem using a menstrual cup  - Participants were more likely to choose the menstrual cup if they were not concerned with manipulation of internal genitalia | - Participants were less likely to choose the menstrual cup if using as a method to reduce menstrual bleeding | - 25.6% reported receiving medical orientation about menstrual cups | - participants concerned about environmental degradation were 6.3x more likely to choose menstrual cup (68.29% of participants cited concern about biodegradation of menstrual hygiene methods as a factor influencing menstrual product choice) |
| **Owen, 2022 / Australia^24^** |  | - One participant had difficulty with mechanics of cup, but wanted to continue using it for its environmental benefits  - One participant had considerable anxiety about insertion, difficulty with extraction, and feared the cup would cause internal damage, but when she did succeed, she loved the sense of "freedom" it gave her, and wanted to continue using it for its environmental benefits  - Learning process was significant but by 3rd menstrual cycle most able to use easy  - Participants reported ease of use of the menstrual cup in workplace and how it minimised the impact of menstruation during the working day: does not require changing, no need to carry replacement products around eg "the stress of making sure that you've got stuff in your bag", no requirement to undo noisy products in bathroom, no need for disposal  - Menstrual cup gives freedom when "going out at night"  - Menstrual cup was a tool of “forgetting” - it relieved emotional labour associated with menstruation and minimised its impact but also led to vivid remembering when needed to empty, wash and replace the cup  - Menstrual cup results in a more direct encounter with menstrual blood: gave participants more information about their menstrual blood: "how much", "how bloodlike it is", some reported enjoying the extra knowledge, finding it "empowering", others found this uncomfortable |  |  | - Environmental benefit important motivator for trying the menstrual cup  - Some participants used menstrual cup primarily for environmental benefit despite anxiety and difficulty using the cup, eg one participant was nervous to try the cup and still felt it was "foreign" after three cycles, but wanted to continue using it for its environmental benefits |
| **Abraham, 2023 / India^25^** | - 78% (n = 146) unwilling to use a menstrual cup | - 52.7% believed insertion of the cup is painful  - 20.3% believe there is need of backup protection whilst using the menstrual cup  - 30.8% reported fear of using the menstrual cup  - 11.2% believed the menstrual cup can get lost in vagina | - 25.7% believed that the menstrual cup cannot be used with a heavy flow | - 67.4% received no health education about the menstrual cup  - Most (n = 123) had knowledge of cup through social media  - 16.4% reported lack of knowledge about how to use a cup |  |
| **Bhanawat, 2023 / India^26^** | - 76.56% (n = 196/256) willing to buy menstrual cup  - 83.20% (n = 213/256) don't want to opt for a menstrual cup as their sanitary protection method  - 5.86% (n = 15/256) believe menstrual cups are best suited to them  - most common source of knowledge regarding menstrual cup was social media: 37.27% (n = 82/256) | % of participants selecting different benefits or harmful effects:  - no requirement of frequent changing (32.72%, n = 72/256)  - physical activity can easily be done (28.18%, n = 62/256)  - pain (9.09%, n = 20/256)  - discomfort (37.11% n = 95/256)  - leakage (14.84% n = 38/256)  Participants who use the menstrual cup:  - 80% (n = 8/10) reported easy to insert and remove  - 60% (n = 6/10) reported easy to clean  - 80% (n = 8/10) reported leakage issues  - 80% (n = 8/10) felt comfortable to use cup  - 20% (n = 2/10) reported pain  - 10% (n = 1/10) reported discomfort or irritation  - 70% (n = 7/10) expressed desire to continue to use |  | - Hesitancy about using cup due to limited knowledge reported by 27.73%  - 80% (n = 176/256) don't know emptying time  - 64.06% (n = 164/256) don't know how to sterilise cup  - 74.09% (n = 163/256) don’t know shelf life | - 30% (n = 66/256) participants reported 'eco-friendly' as a benefit |
| **James, 2024 / India^27^** | - Health education about menstrual cups resulted in 35% increase in total attitude score towards cup use  - Prior to intervention 18.1% (n = 15/83) considered cups to be safe → increased to 67.5% (n = 56/83) post-education.  - Increase in usage of cups by 6% post-intervention after 1 month. | - 6.02% (n = 5/83) believed cup does NOT cause pain and discomfort at baseline → increased to 33.7% post-education.  - 78.3% (n = 65/83) have fear of menstrual cup insertion causing hymen tear → decreased to 67.5% post-education.  - 85.8% (n = 71/83) concerned about leakage → increased to 86.7% (n = 72/83) post-education.  - Education about cup resulted in 27.7% reduction in reported fear of pain and discomfort |  | - Health education about menstrual cups resulted in 42% increase in awareness about menstrual cup use  - Some reported belief that cup can be used as a contraceptive method | - At baseline 77.1% (n = 64/19) were unaware that menstrual cups are a sustainable product |
| **Soumyaja, 2024 / India^28^** | - Mean value for intention to use a menstrual cup was 3.31/5 (‘average’ intention to use) | Of those not using the menstrual cup regularly or occasionally:  - 56% (n = 952/1700) fear of vaginal insertion  - 21% (n = 357/1700) fear of menstrual cup getting stuck in vagina  - 5% (n = 85/1700) fear of leakage  Of those using the menstrual cup regularly or occasionally:  - 45% (n = 67/148) menstrual cup is convenient |  | Of those who were not using cups regularly or occasionally, up to 15% identified not having heard of the cup as a factor hindering use | Of those using the menstrual cup regularly or occasionally:  - 24% (n = 36/148) reported sustainability / concern for the environment as a factor influencing adoption of the menstrual cup |

| **Lead Author, Year / Country** | **Availability of reusable products** | **Financial concern** | **Concerns about physical health** | **Sociocultural influences** | **Comparison of use with single-use products** |
| --- | --- | --- | --- | --- | --- |
| **Grose, 2014 / USA^19^** |  | Only 13.2% said would be somewhat or very likely to buy the menstrual cup if priced within their budget |  | - Higher levels of self-objectification directly predicted more negative attitudes towards periods, which in turn, directly predicted negative ratings for likelihood of buying cup, using cup, and overall negative reaction to cup  - Self-objectification did not explain negative ratings for likelihood of recommending cup and perceived quality of cup, i.e. no significant indirect relationship between self-objectification, and these two measures  - No differences for attitudes towards own menstruation or ratings of the menstrual cup between ethnicities  - Increasing age was related to positivity toward the menstrual cup |  |
| **Huang, 2019 / Taiwan^20^** |  | Students reported higher menstrual cup use intention if they believed it would save lots of money |  | - Bisexuals were nearly twice as likely (AOR = 1.78) than straight girls to have high menstrual cup use intention  - Lesbians were 69% less likely than straight girls to have high menstrual cup use intention | - 31.6% of students who mainly used pads had high menstrual cup use intention  - 61.4% of students mainly using tampons had high menstrual cup use intention |
| **Beksinska, 2021 / South Africa^21^** | At follow up, loss of the cup was reported as a reason for not having tried it | 62.3% of participants accepted the cup at baseline due to financial benefit |  | Concerns about virginity were reported as a reason for not having tried the menstrual cup | - Across all follow-up points ⅔-¾ of students reported preferring menstrual cup to their usual menstrual hygiene products  - 49.3% of students accepted the menstrual cup at baseline due to a desire to try a new method |
| **Ganz, 2022 / South Africa^22^** |  | 85.31% agree/strongly agree that the menstrual cup is a more cost-effective alternative | 78.04% disagreed that the menstrual cup is unhygienic and unsafe |  |  |
| **Lobascz, 2022 / Brazil^23^** |  |  | Participants concerned about vaginal health were twice as likely to choose menstrual cup |  |  |
| **Owen, 2022 / Australia^24^** |  | - Almost all participants believed the cup is cost-effective, coupling the environmental impact with economic worthiness  - "so much better to not be spending lots of money every month on single use products"  - "nice to not have to keep buying tampons all the time"  - One participant linked it with charitable outreach efforts to 'deprived' geographical areas | - One participant was surprised that use of the menstrual cup led to no longer having previously debilitating menstrual pain | - Cup was considered to be “cool”  - Cup challenged participants to think and talk about menstruation more and appeared to legitimise conversation about menstruation with partners, colleagues, family and friends  - For one participant, feminist concerns outweighed sustainability as a motivation for trying the cup, others broadly referenced feminism as a contributing factor  - Belonging to feminist peer groups was cited as a reason for wanting to try the cup |  |
| **Abraham, 2023 / India^25^** | - 11% reported lack of availability of cup |  | - 20.3% believe long-term use of cup can cause infections of reproductive tract  - 11.8% believe cup can cause UTIs  - 6.4% believe cup can cause irregular cycles | - 16.4% (n = 31) willing to use cup after marriage  - 9.6% reported conventional beliefs as a reason not to use cup  - 7.5% believed cup can cause loss of virginity | - 17.8% reported satisfaction with current method of menstrual hygiene management as a reason to not switch to a reusable product |
| **Bhanawat, 2023 / India^26^** |  | - 23.63% (n = 52/256) participants reported 'economical' as a benefit | % of participants citing different consequences of using the cup:  - urinary problems (11.81% n = 26/256)  - infection (18.63% (n = 41/256)  - allergies (6.25% n = 16/256)  - rash-free periods (37.27%, n = 82/256)  - itch-free periods (32.27%, n = 71/256) |  | - 90.23% (n = 231/256) believe sanitary pads are best suited for them  - 1.17% (n = 3/256) believe sanitary tampons are best suited for them  - 83.2% (n = 196/256) don’t want to change current method of menstrual hygiene management |
| **James, 2024 / India^27^** |  |  | - 84.3% (n = 70/83) believe cup can cause allergies.  - 91.6% (n = 76/83) thought urination affected whilst using cup  - 18.1% (n = 15/83) believed cup is a safe device | - 30.1% (n = 25/83) believe cups can be used by unmarried women  - 14.5% (n = 12) believe cups don't result in loss of virginity | - 25.3% (n = 21/83) believed cups are better than sanitary napkins at baseline  - After intervention increased to 61.4% (n = 51/83). |
| **Soumyaja, 2024 / India^28^** |  | Of those using the menstrual cup regularly or occasionally:  - 6% (n = 9/148) reported low-cost as a factor influencing adoption of the menstrual cup | Of those using cup regularly/occasionally:  - 25% (n = 37/148) reported health reasons as a factor influencing adoption of cup  Of those not using cup regularly/occasionally:  - 3% (n = 51/1700) fear of infection |  |  |
