# Supplementary material for: Experiences, perceptions and barriers to use of reusable menstrual products among university students globally: a systematic review
Source: BMJ Open. 2025 Aug 6;15(8):e103159. doi: 10.1136/bmjopen-2025-103159 (PMC12336580; doi:10.1136/bmjopen-2025-103159)
Supplement: online supplemental file 4 [file bmjopen-15-8-s004.docx]

GRADE-CERQual Evidence Profile

| Summary of review finding | Studies contributing to the review finding | Methodological limitations | Coherence | Adequacy | Relevance | CERQual assessment of confidence in the evidence | CERQual assessment of confidence in the evidence |
| --- | --- | --- | --- | --- | --- | --- | --- |
| 1. Financial Implications of reusable menstrual products |  |  |  |  |  |  |  |
| No included studies reported negative views about financial implications. Four studies found participants viewed the menstrual cup as cost-effective, motivating its use. Three studies found less than a quarter of participants perceived this as an important benefit. | 19, 20, 21, 22, 24, 26, 28 | Moderate concerns: 2 studies with no or very minor issues, 4 study with minor issues (recruitment, risk of non-response bias, data validation), 1 study with substantial issues (lack of integration of qualitative and quantitative data) | No or very minor concerns | Minor concerns: 7 studies contributed to this finding, of which 4 had a large number of participants and perspectives | Minor concerns: 2 of the 7 studies have partial relevance as only assessed medical students and not university students as a whole | **Moderate confidence** | Moderate concerns regarding methodological limitations (with 1 study with substantial issues in use of mixed-methods data). No, very minor, or minor concerns regarding coherence, adequacy and relevance. |
| 2. Environmental impact of reusable menstrual products |  |  |  |  |  |  |  |
| The proportion of participants reporting sustainability as an important motivator of menstrual product use varied significantly. One study also reported low awareness of sustainability as a benefit. | 23, 24, 26, 27, 28 | Minor concerns: 2 studies with no or very minor issues, 3 study with minor issues (recruitment, risk of non-response bias, data validation) | No or very minor concerns | Minor concerns: Only 5 studies contributed to this finding, however the review finding is primarily descriptive and relatively superficial data was judged to be sufficient in assessing adequacy | Minor concerns: 2 of the 7 studies have partial relevance as only assessed medical students and not university students as a whole | **High confidence** | 5 studies with minor concerns about methodological limitations, adequacy and relevance. No or very minor concerns about coherence. |
| 3. Practical concerns, Education and knowledge about reusable menstrual products |  |  |  |  |  |  |  |
| 3.1 Formal education about reusable menstrual products was low. | 23, 25 | Minor concerns: 1 study with no or very minor issues, 1 study with minor issues (risk of non-response bias) | No or very minor concerns | Serious concerns: While the finding was relatively narrow in scope, only 2 studies contributed to this finding and both offered thin data | Minor concerns: Both studies have partial relevance as only assessed either medical students, or engineering and arts students, and not university students as a whole | **Low confidence** | Only 2 studies with serious concerns regarding adequacy of data, despite only having no, very minor, or minor concerns regarding methodological limitations, coherence or relevance. |
| 3.2 Practical concerns (such as leakage, insertion, removal, discomfort, changing away from home, direct encounter with menstrual blood) were frequently cited. However, other participants found the menstrual cup convenient and easy to use. | 19, 20, 21, 22, 23, 24, 25, 26, 27, 28 | Moderate concerns: 3 studies with no or very minor issues, 6 studies with minor issues (recruitment, risk of non-response bias, data validation), 1 study with substantial issues (lack of integration of qualitative and quantitative data) | No or very minor concerns | Minor concerns: All 10 studies contributed to information about practical concerns, however data were of varying richness | No or very minor concerns: All ten studies contributed to information about practical concerns and represented 6 countries (USA, Taiwan, South Africa, Brazil, Australia, India) | **Moderate confidence** | Moderate concerns regarding methodological limitations (with 1 study with substantial issues in use of mixed-methods data). No, very minor, or minor concerns regarding coherence, adequacy and relevance. |
| 3.3 Lack of knowledge and experience was linked to negative perceptions. Increased knowledge or awareness, provision of education and/or a reusable product increased positive perceptions of reusable menstrual products. | 19, 20, 21, 26, 27, 28 | Moderate concerns: 1 studies with no or very minor issues, 4 studies with minor issues (recruitment, risk of non-response bias, data validation), 1 study with substantial issues (lack of integration of qualitative and quantitative data) | No or very minor concerns | Minor concerns: 6 studies contributed to this finding, of 4 had a large number of participants and perspectives | Minor concerns: Only 1 of the 6 studies has partial relevance as only assessed medical students and not university students as a whole | **Moderate confidence** | Moderate concerns regarding methodological limitations (with 1 study with substantial issues in use of mixed-methods data). No, very minor, or minor concerns regarding coherence, adequacy and relevance. |
| 4. Concerns about physical health and safety |  |  |  |  |  |  |  |
| Reported impacts of the menstrual cup on physical health include both positive and negative impacts: promotion of vaginal health, rash- and itch-free periods, urinary problems, infections and allergies. This meant concerns about physical health were both a barrier and motivator to use, depending on individual participants' views. | 22, 23, 25, 26, 27, 28 | Moderate concerns: 2 studies with no or very minor issues, 4 studies with minor issues (recruitment, risk of non-response bias, data validation) | No or very minor concerns | Minor concerns: 6 studies contributed to this finding, and although some studies have fewer participants, the review finding is primarily descriptive and relatively superficial data was judged to be sufficient in assessing adequacy | Moderate concerns: 4 of the 6 studies have partial relevance as only assessed either medical students, or engineering and arts students, and not university students as a whole | **Moderate confidence** | Moderate concerns regarding methodological limitations (with 1 study with substantial issues in use of mixed-methods data) and relevance (with students not entirely representative of population of university students). No, very minor, or minor concerns regarding coherence and adequacy. |

GRADE-CERQual Summary of Findings

| Summary of review finding | Studies contributing to the review | CERQual assessment of confidence in the evidence | Explanation of CERQual assessment |
| --- | --- | --- | --- |
| 1. Financial benefit of reusable menstrual products was a motivator of use for some, but not all participants. There were no reports of negative views about financial implications. | 19, 20, 21, 22, 24, 26, 28 | **Moderate confidence** | Moderate concerns regarding methodological limitations (with 1 study with substantial issues in use of mixed-methods data). No, very minor, or minor concerns regarding coherence, adequacy and relevance. |
| 2. The proportion of participants reporting sustainability as an important motivator of menstrual product use varied significantly and not all participants were aware of sustainability as a benefit | 23, 24, 26, 27, 28 | **High confidence** | 5 studies with minor concerns about methodological limitations, adequacy and relevance. No or very minor concerns about coherence. |
| 3.1 Formal education about reusable menstrual products was low | 23, 25 | **Low confidence** | Only 2 studies with serious concerns regarding adequacy of data, despite only having no, very minor, or minor concerns regarding methodological limitations, coherence or relevance. |
| 3.2 Many varied practical concerns about using reusable menstrual products were frequently mentioned, although other participants found the menstrual cup easy and convenient to use | 19, 20, 21, 22, 23, 24, 25, 26, 27, 28 | **Moderate confidence** | Moderate concerns regarding methodological limitations (with 1 study with substantial issues in use of mixed-methods data). No, very minor, or minor concerns regarding coherence, adequacy and relevance. |
| 3.3 Lack of knowledge and experience with reusable products was associated with negative perceptions and education and/or provision of a reusable menstrual product increased positive perceptions | 19, 20, 21, 26, 27, 28 | **Moderate confidence** | Moderate concerns regarding methodological limitations (with 1 study with substantial issues in use of mixed-methods data). No, very minor, or minor concerns regarding coherence, adequacy and relevance. |
| 4. Participants reported both negative and positive perceptions about the influence of reusable menstrual products on physical health | 22, 23, 25, 26, 27, 28 | **Moderate confidence** | Moderate concerns regarding methodological limitations (with 1 study with substantial issues in use of mixed-methods data) and relevance (with students not entirely representative of population of university students). No, very minor, or minor concerns regarding coherence and adequacy. |

References:

1. Grose RG, Grabe S. Sociocultural Attitudes Surrounding Menstruation and Alternative Menstrual Products: The Explanatory Role of Self-Objectification. Health Care for Women International 2014 Jun; 3;35(6):677-94. Available from: <https://doi.org/10.1080/07399332.2014.888721>
2. Huang PT, Huang JH. Menstrual cup use intention and the moderating effects of sexual orientation and gender characteristic among female university students in Taiwan: a theory-driven exploration. Archives of Sexual Behavior. 2020 May;49(4):1355-66. Available from: <https://doi.org/10.1007/s10508-019-1412-y>
3. Beksinska M, Nkosi P, Zulu B, et al. Acceptability of the menstrual cup among students in further education institutions in KwaZulu-Natal, South Africa. The European Journal of Contraception & Reproductive Health Care. 2021 Jan 2;26(1):11-6. Available from: <https://doi.org/10.1080/13625187.2020.1815005>
4. Ganz C, Lever E, Bredenkamp J, et al. The Understanding and Perception of the Menstrual Cup Among Medical Students. The Journal of Obstetrics and Gynecology of India. 2022 Oct;72(5):439-45. Available from: <https://doi.org/10.1007/s13224-022-01661-8>
5. Lobascz BC, Reis MB, Mendes GD, et al. Determinants of menstrual cup use among undergraduate medical students: A cross‐sectional study. International Journal of Gynecology & Obstetrics. 2023 Mar;160(3):1007-11. Available from: <https://doi.org/10.1002/ijgo.14450>
6. Owen L. Stigma, sustainability, and capitals: A case study on the menstrual cup. Gender, Work & Organization. 2022 Jul;29(4):1095-112. Available from: <https://doi.org/10.1111/gwao.12808>
7. Abraham R, Rajan MP, Sajithamony, et al. Knowledge, Acceptability and Misconceptions Regarding Menstrual Cup among College Students of Kerala: A Cross-Sectional Study. Indian Journal of Public Health Research & Development. 2023 Mar 15;14(2):399-406. Available from: <https://doi.org/10.37506/ijphrd.v14i2.19139>
8. Bhanawat M, Mehta D, Vachhani P, et al. A Cross-Sectional Assessment of Knowledge, Attitude and Practice Regarding Menstrual Cup. International Journal of Toxicological and Pharmacological Research 2023; 13(10); 176-183. Available from: <https://impactfactor.org/PDF/IJTPR/13/IJTPR,Vol13,Issue10,Article34.pdf>
9. James C, Binu DM, Regy MM, et al. The impact of health education intervention on perceptions of menstrual cup among college going female students in peri-urban Bangalore. International Journal of Adolescent Medicine and Health. 2024 Jun 27;36(3):279-84. Available from: <https://doi.org/10.1515/ijamh-2023-0185>
10. Soumyaja. Sustainable menstruation and menstrual cups: Study on awareness and intention to use in the state of Kerala. Environment and Social Psychology. 2024;9(8):2743. Available from: <https://doi.org/10.59429/esp.v9i8.2743>
