# Supplementary material for: Experiences, perceptions and barriers to use of reusable menstrual products among university students globally: a systematic review
Source: BMJ Open. 2025 Aug 6;15(8):e103159. doi: 10.1136/bmjopen-2025-103159 (PMC12336580; doi:10.1136/bmjopen-2025-103159)
Supplement: Supplementary file 5 [file bmjopen-2025-103159.reviewer_comments.pdf]

PEER REVIEW HISTORY

BMJ Open publishes all reviews undertaken for accepted manuscripts. Reviewers are asked to complete a checklist review form and are provided with free text boxes to elaborate on their assessment. These free text comments are reproduced below.

ARTICLE DETAILS

Title (Provisional)

Experiences, perceptions and barriers to use of reusable menstrual products among university students globally: a systematic review

Authors

Johnson, Emma; Seed, Lydia; Biju, Ashna; Tulinius, Charlotte

VERSION 1 - REVIEW

**Reviewer**	**1**
**Name**	**Bannow, Bethany Samuelson**
**Affiliation**	**Oregon Health & Science University**
**Date**	**03-May-2025**
**COI**	**None**

Introduction

The need for this study in a medical publication is not clear from this introduction – while increasing use of reusable products is certainly better for the environment, there is no known medical benefit. Unless a medical case can be made for this investigation it is considerably better suited to an environmental journal.

Results

Data quality assessment should be included, at least in part, in the results section and should not be limited to the supplementary material alone.

Discussion

Implications of data quality should be addressed in the discussion as well

The authors briefly mention period product insecurity in university students in the discussion, and the potential barrier lack of menstrual products presents to education in the introduction. Do the authors intend to suggest that increased use of menstrual cups would reduce this barrier? If so this needs to be stated explicitly and a case needs to be made – is there any evidence that menstrual cup use increases ability to attend school?

Reviewer 2

Name Panda, Bhuputra

Affiliation Public Health Foundation of India, Indian Institute of Public Health Bhubaneswar

Date 05-May-2025

COI None

Review comments for the manuscript

Comments to the author - The manuscript addresses an important and timely topic with well-structured sections. However, the introduction could be strengthened by briefly referencing global trends and more clearly defining the study's scope and novelty—particularly specifying which reusable products and the geographic focus. The objective would benefit from being more concise and direct. In the results section, ensure all key claims are properly cited consider revising the entire manuscript for better clarity. These revisions will enhance the overall clarity, relevance, and impact of the study.

**Introduction**	The introduction provides a strong foundation for the topic; however, including brief references to global initiatives or trends in other countries (such as India, Kenya, or Australia) would help establish the broader relevance of the issue and offer a comparative context. Add a sentence early on to underscore why menstrual hygiene management (MHM) is not just a personal health issue but a broader public health, environmental, and gender equity issue. Also, the final paragraph introduces the study's purpose but could more precisely define the novelty and scope of the research, particularly by specifying which reusable products are being studied and the geographic focus (e.g., university students in the UK).<br>Consider refining the objective for greater clarity and conciseness. If the literature review supports a global gap in this area, stating this explicitly would further strengthen the rationale for conducting the study.
**Methodology**	**Clarification of MMAT Use**: The MMAT is specifically designed to assess different types of study designs—qualitative, quantitative (including descriptive, randomized, and non-randomized), and mixed methods—within a single, integrated tool. Therefore, it is unnecessary and potentially confusing to state that separate tools within the MMAT were used for different study types. This phrasing should be revised for clarity.<br>**Referencing**: The authors should ensure that the MMAT and any other mentioned appraisal tools (even if not ultimately used) are properly referenced.

**Result**	The result section is well-written and presents the findings clearly. However, a reference should be added to support the statement in the fourth paragraph on page 8. Please ensure that all key claims throughout the manuscript are appropriately cited. Also, the fifth paragraph could be framed a little better for, more clarity and more precise articulation of the findings.
**Discussion**	The discussion section is well-structured; however, some points could strengthen this further. The financial benefit range ("6% to 85.31%") on page number 9-line number 11 appears precise but lacks contextual explanation. Consider clarifying why this range is so broad (e.g., differences in study populations or definitions). There is repetition around **practical experience reducing concerns** and **education improving perceptions**. These points are important but can be presented more concisely.
**Conclusion**	The author might consider including a brief statement on how universities and public health bodies can implement these findings through policy changes, such as ensuring inclusive menstrual health initiatives and infrastructure (e.g., private washing facilities) in this section. They can further highlight the importance of context-specific strategies, particularly in low- and middle-income countries, where affordability, taboos, and access pose unique challenges. Adding a note on the need for standardized outcome measures in future studies would address current inconsistencies in how perceptions and usage are reported.

VERSION 1 - AUTHOR RESPONSE

Reviewer: 1
Dr. Bethany Samuelson Bannow, Oregon Health & Science University
Comments to the Author:

Introduction:

- The need for this study in a medical publication is not clear from this introduction – while increasing use of reusable products is certainly better for the environment, there is no known medical benefit. Unless a medical case can be made for this investigation it is considerably better suited to an environmental journal.
The first paragraph of the introduction has been edited to highlight the broader issues associated with menstrual hygiene management, including public and personal health. A sentence has also been added to the end of this paragraph expanding on these issues, highlighting medical issues associated with poor menstrual hygiene management e.g. reproductive and urinary tract infections and mental health problems.

Results:

- Data quality assessment should be included, at least in part, in the results section and should not be limited to the supplementary material alone.
A new subsection has been added to the results section between 'Knowledge and patterns of usage of reusable menstrual products' and 'Perceptions and determinants of use of reusable menstrual products', entitled 'Quality Assessment and Confidence in Review Findings'. In this section we have now provided more detail about the findings of the MMAT and how quality assessment impacted our selection of studies for the main synthesis, as per the SWiM checklist.

Discussion:

- Implications of data quality should be addressed in the discussion as well
We have added a sentence to the first paragraph of the discussion, summarising the findings of assessment of methodological quality and the certainty of the findings, and what the implications are of this. For the finding with low certainty (low levels of formal education), due to low adequacy of data, we have included a statement to highlight this uncertainty (paragraph 4).

- The authors briefly mention period product insecurity in university students in the discussion, and the potential barrier lack of menstrual products presents to education in the introduction. Do the authors intend to suggest that increased use of menstrual cups would reduce this barrier? If so this needs to be stated explicitly and a case needs to be made – is there any evidence that menstrual cup use increases ability to attend school?
Current research assessing the impact of reusable menstrual products on school attendance is inconsistent, so there is not strong evidence that provision of reusable products reduce the barrier to education presented by period poverty. Two sentences have been added to the third paragraph of the introduction addressing this to make it clear that there is not currently evidence for this claim and that we are therefore calling for more research into this area, rather than stating that this is a known benefit of reusable products. In the discussion, period product insecurity is mentioned in the context of concerns about upfront costs of reusable products and their potential for cost-savings over time, rather than in the context of improving access to education.

Reviewer: 2
Dr. Bhuputra Panda, Public Health Foundation of India
Comments to the Author:
Pls refer the attached file for further details.
See below

Comments to the author: The manuscript addresses an important and timely topic with well- structured sections. However, the introduction could be strengthened by briefly referencing global trends and more clearly defining the study's scope and novelty—particularly specifying which reusable products and the geographic focus. The objective would benefit from being more concise and direct. In the results section, ensure all key claims are properly cited consider revising the entire manuscript for better clarity. These revisions will enhance the overall clarity, relevance, and impact of the study.

See detailed response below

Introduction:

- The introduction provides a strong foundation for the topic; however, including brief references to global initiatives or trends in other countries (such as India, Kenya, or Australia) would help establish the broader relevance of the issue and offer a comparative context.
Further references have been added into the introduction, highlighting global trends and initiatives. We have now referenced a variety of governments which provide free menstrual product provision in schools (including those in Australia and Kenya), as well as global trends in the reusable menstrual product market and of their use in India.

- Add a sentence early on to underscore why menstrual hygiene management (MHM) is not just a personal health issue but a broader public health, environmental, and gender equity issue.
The first paragraph of the introduction has been edited to highlight the broader issues associated with menstrual hygiene management, including public health, environmental, gender equality and personal health. A sentence has also been added to the end of this paragraph expanding on these issues, mentioning missed educational opportunities, stigma and inequality, medical issues and plastic waste.

- Also, the final paragraph introduces the study's purpose but could more precisely define the novelty and scope of the research, particularly by specifying which reusable products are being studied and the geographic focus (e.g., university students in the UK).
The final paragraph has been revised to specify the population more clearly (university students and those in higher education), the products being studied (all reusable menstrual products with a focus on menstrual cups) and the geographic focus (a global perspective).

- Consider refining the objective for greater clarity and conciseness. If the literature review supports a global gap in this area, stating this explicitly would further strengthen the rationale for conducting the study.
In the final paragraph of the introduction we have now stated more explicitly that the literature review supports a global gap in this area. We have also refined the objective in this paragraph in line with refinement of the title as above. We have expanded 'perceptions' to state that we are investigating knowledge, usage patterns, perceptions, and determinants of use of reusable menstrual products, particularly menstrual cups, among university and higher education students globally.

Methodology:

- Clarification of MMAT Use: The MMAT is specifically designed to assess different types of study designs—qualitative, quantitative (including descriptive, randomized, and non-randomized), and mixed methods—within a single, integrated tool. Therefore, it is unnecessary and potentially confusing to state that separate tools

within the MMAT were used for different study types. This phrasing should be revised for clarity.
The description of our use of the MMAT has been revised so that the separate tools within the MMAT are now not mentioned. The phrasing now states that the MMAT was used to assess the quality of the included studies without extra detail.

- Referencing: The authors should ensure that the MMAT and any other mentioned appraisal tools (even if not ultimately used) are properly referenced.
The quality assessment section of the methods has been revised so that the MMAT and CASP tools are both appropriately referenced. The additional SWiM and GRADE-CERQual tools which have now been included are also appropriately referenced.

<u>Results:</u>

- The result section is well-written and presents the findings clearly. However, a reference should be added to support the statement in the fourth paragraph on page 8.
References have been added to support the statements about perceptions of the influence of the menstrual cup on physical health, which is the 4th paragraph of the results section.

- Please ensure that all key claims throughout the manuscript are appropriately cited.
The manuscript has been reviewed and any key claims which were not appropriately cited now have appropriate citations.

- Also, the fifth paragraph could be framed a little better for, more clarity and more precise articulation of the findings.
The fifth paragraph has been revised for clarity, with a clearer summarising sentence at the start, followed by more concise grouping of results. The wording has also been revised to ensure the findings are clearly articulated and ambiguous wording is removed.

<u>Discussion:</u>

- The discussion section is well-structured; however, some points could strengthen this further. The financial benefit range ("6% to 85.31%") on page number 9-line number 11 appears precise but lacks contextual explanation. Consider clarifying why this range is so broad (e.g., differences in study populations or definitions).
A sentence has been added after this statement to suggest reasons for this potential discrepancy, including variation in experience of participants with the menstrual cup and the lack of standardised measure of financial benefit across the studies.

- There is repetition around practical experience reducing concerns and education improving perceptions. These points are important but can be presented more concisely.
The two paragraphs discussing practical experience and education have been moved in the discussion so they are next to each other, allowing much of the repeated information to be cut and articulated more concisely, emphasising the

overarching point linking increased experience, either through practical experience or education, improves perceptions towards the menstrual cup.

Conclusion:

- The author might consider including a brief statement on how universities and public health bodies can implement these findings through policy changes, such as ensuring inclusive menstrual health initiatives and infrastructure (e.g., private washing facilities) in this section.
We have added statements to the conclusion to provide more clarity on policy change to increase uptake of reusable products, including inclusive educational initiatives and menstrual product provision programmes, and appropriate infrastructure eg bathrooms with private washing facilities

- They can further highlight the importance of context-specific strategies, particularly in low- and middle-income countries, where affordability, taboos, and access pose unique challenges.
A sentence has been added to the final paragraph of the conclusion, highlighting the importance of context-specific strategies to ensure efficacy of implemented programmes as cultural differences, affordability and access to both menstrual products and washing facilities are all essential considerations.

- Adding a note on the need for standardized outcome measures in future studies would address current inconsistencies in how perceptions and usage are reported.
A sentence has been added to the end of the final paragraph in the conclusion to emphasise the need for these standardised outcome measures in further research.
